# Supplementary material for: Influence of pharmacists and infection control teams or antimicrobial stewardship teams on the safety and efficacy of vancomycin: A Japanese administrative claims database study
Source: PLoS One. 2022 Sep 9;17(9):e0274324. doi: 10.1371/journal.pone.0274324 (PMC9462795; doi:10.1371/journal.pone.0274324)
Supplement: S1 Table — TDM, therapeutic drug monitoring; AEDs, antiepileptic drugs; ICT, infection control team; AST, antimicrobial stewardship team. (DOCX) [file pone.0274324.s001.docx]

**S1 Table.** **Reimbursement and Claimed Requirements**

|  | Reimbursement | Claimed requirements |
| --- | --- | --- |
| TDM | Treatment and management fee for specific drugs | Measurement of blood concentration and precise dosage control when patients are administered specific drugs, such as glycopeptides, certain immunosuppressive drugs, or AEDs. |
| Pharmacist intervention | Inpatient pharmaceutical services premium | Implementation of pharmacotherapy contributing to the enhancement of efficacy and safety in hospital wards by pharmacists for all hospitalized patients in the ward |
|  | Drug management and guidance fee | Implementation of drug administration guidance and pharmacological management for each hospitalized patient, such as evaluating the dosage, route of administration, dosing rate, and drug-drug interaction |
| Infectious Disease- associated team intervention | Infection prevention and control premium | Implementation of systematic infectious disease control measures with ICT, such as monitoring of nosocomial infections, appropriate use of antimicrobial agents, and infection prevention for medical staff |
|  | Antimicrobial stewardship support premium | Promotion of appropriate use of antimicrobial agents by AST at medical institutions where infection prevention and control premium is calculated |

TDM, therapeutic drug monitoring; AEDs, antiepileptic drugs; ICT, infection control team; AST, antimicrobial stewardship team.
