# Supplementary material for: Influence of pharmacists and infection control teams or antimicrobial stewardship teams on the safety and efficacy of vancomycin: A Japanese administrative claims database study
Source: PLoS One. 2022 Sep 9;17(9):e0274324. doi: 10.1371/journal.pone.0274324 (PMC9462795; doi:10.1371/journal.pone.0274324)
Supplement: S2 Table — * “Number” indicates the number of patients who used the drug concerned concomitantly within 7 days from the index date, defined as the date of vancomycin initiation. (DOCX) [file pone.0274324.s002.docx]

**S2 Table. Number of patients who claimed treatment and management fee for specific drugs excluding vancomycin**

| **Drug Name** | **Number^＊^** | **Drug Name** | **Number^＊^** |
| --- | --- | --- | --- |
| **Digitalis preparation** | | **Antiarrhythmic agents** | |
| Digoxin | 1362 | Amiodarone hydrochloride | 1627 |
| Methyldigoxin | 299 | Aprindine hydrochloride | 180 |
| **Antiepileptic drugs** | | Bepridil hydrochloride hydrate | 169 |
| Carbamazepine | 871 | Cibenzoline succinate | 279 |
| Fosphenytoin | 521 | Disopyramide Disopyramide phosphate | 215 |
| Phenobarbital Phenobarbital sodium | 785 | Flecainide acetate | 75 |
| Phenytoin Phenytoin sodium | 1709 | Mexiletine hydrochloride | 263 |
| Phenytoin-phenobarbital combination | 27 | Pilsicainide hydrochloride hydrate | 524 |
| Sodium valproate | 1719 | Pirmenol hydrochloride hydrate | 5 |
| **Immunosuppressive drug** | | Procainamide | 66 |
| Ciclosporin | 428 | Propafenone hydrochloride | 32 |
| Everolimus | 14 | Quinidine sulfate hydrate | 5 |
| Mycophenolate mofetil | 128 | Sotalol hydrochloride | 45 |
| Tacrolimus hydrate | 928 | **Theophylline preparation** | |
| **Aminoglycoside antibiotic** | | Theophylline | 874 |
| Amikacin sulfate | 920 | **Lithium preparation** | |
| Arbekacin sulfate | 304 | Lithium carbonate | 83 |
| Gentamicin sulfate | 1952 | **Imatinib** | |
| Tobramycin | 167 | Imatinib mesylate | 46 |
| **Glycopeptide antibiotic** | | **Sirolimus preparation** | |
| Teicoplanin | 666 | Sirolimus | 0 |
| **Triazole antifungal agent** | | **Sunitinib** | |
| Voriconazole | 1361 | Sunitinib malate | 0 |

* “Number” indicates the number of patients who used the drug concerned concomitantly within 7 days from the index date, defined as the date of vancomycin initiation.
