# Supplementary material for: Influence of pharmacists and infection control teams or antimicrobial stewardship teams on the safety and efficacy of vancomycin: A Japanese administrative claims database study
Source: PLoS One. 2022 Sep 9;17(9):e0274324. doi: 10.1371/journal.pone.0274324 (PMC9462795; doi:10.1371/journal.pone.0274324)
Supplement: S3 Table — These codes were defined based on the Various Information of Medical Fee operated by the Ministry of Health, Labour and Welfare (https://shinryohoshu.mhlw.go.jp/). MRCNS, methicillin-resistant coagulase-negative staphylococci. (DOCX) [file pone.0274324.s003.docx]

**S3 Table.** **Codes and diagnostic information for classification of infection sites**

| **Site of Infection** | **Japanese Disease Code** | | | | | |
| --- | --- | --- | --- | --- | --- | --- |
| Respiratory infection | 4824001 | 4829003 | 4860018 | 4860030 | 4860043 | 5070002 |
|  | 5070003 | 5109010 | 5109015 | 5109016 | 8830121 | 8830122 |
|  | 8832177 | 8833161 | 8834801 | 8834867 | 8838214 | 8838416 |
|  | 8838435 | 8838600 | 8838823 | 8838876 | 8839831 | 8841219 |
|  | 8844803 | 8847878 | 9973011 |  |  |  |
| Bacteremia/sepsis | 381001 | 389004 | 389012 | 389016 | 7718002 | 7718005 |
|  | 7855015 | 7907001 | 8830124 | 8830966 | 8832182 | 8834106 |
|  | 8835355 | 8838817 | 8838818 | 8838821 | 8838822 | 8838824 |
|  | 8839900 | 8841001 | 8841002 | 8841319 | 8847009 | 8847862 |
| Infective endocarditis | 4210002 | 4210003 | 4210005 | 4210009 | 8830116 | 8830176 |
|  | 8832314 | 8832359 | 8832388 | 8834111 | 8835117 | 8836077 |
|  | 8836697 | 8838820 |  |  |  |  |
| Skin and soft tissue infections | 6860021 | 6869044 | 7854026 | 8830118 | 8835073 | 8836665 |
|  | 8838755 | 8845681 |  |  |  |  |
| Bone and joint infections | 5249004 | 5264004 | 5264024 | 7110005 | 7166019 | 7209002 |
|  | 7209005 | 7300002 | 7300008 | 7301002 | 7301011 | 7301012 |
|  | 7301014 | 7301015 | 7301017 | 7301018 | 7302002 | 7302004 |
|  | 7302007 | 7302008 | 7302010 | 7302012 | 7302013 | 7302016 |
|  | 7302020 | 7302024 | 7302025 | 7302036 | 7302038 | 7302042 |
|  | 7302043 | 7302044 | 7302045 | 7302046 | 7302047 | 7302048 |
|  | 7302049 | 7302050 | 7303012 | 8830115 | 8830117 | 8830183 |
|  | 8831044 | 8831321 | 8831415 | 8831525 | 8831619 | 8831910 |
|  | 8832304 | 8832332 | 8832335 | 8832547 | 8832953 | 8833051 |
|  | 8833099 | 8833100 | 8833101 | 8833218 | 8833349 | 8833804 |
|  | 8833939 | 8834419 | 8834507 | 8835337 | 8836014 | 8837308 |
|  | 8838819 | 8839458 | 8839825 | 8840169 | 8840312 | 8840328 |
|  | 8840340 | 8840367 | 8840816 | 8842142 | 8842143 | 8842144 |
|  | 8842145 | 8842146 | 8842147 | 8842148 | 8842149 | 8842339 |
|  | 8842473 | 8842475 | 8842504 | 8842535 | 8843286 | 8843613 |
|  | 8846253 | 8846268 | 8847072 | 8847073 | 8847085 | 8847088 |
|  | 8847090 | 8847091 | 8847140 | 8847141 | 8847556 | 8847557 |
|  | 8847832 | 8848439 | 8848558 | 8848673 | 8848849 | 8848850 |
|  | 8849020 | 8849021 | 8849048 | 8849049 |  |  |
| Intra-abdominal infections | 5672005 | 5672025 | 5678003 | 5679005 | 5679006 | 5679012 |
|  | 5679015 | 5720005 | 5720007 | 8830125 | 8831407 | 8835358 |
|  | 8835360 | 8836093 | 8839029 | 8839653 |  |  |
| Central nervous System infections | 408004 | 3203001 | 3209004 | 3216012 | 3229007 | 3241001 |
|  | 3249001 | 3249002 | 8830119 | 8831417 | 8842119 |  |
| Urinary tract infections | 389011 | 5990009 | 8832418 | 8835056 | 8838561 | 8839047 |
|  | 8839875 | 8840382 | 8842093 | 8848848 |  |  |
| Febrile neutropenia | 8842350 |  |  |  |  |  |
| MRCNS infection | 8833325 | 8847069 | 8847070 | 8847071 | 8847864 |  |
| Others | 381003 | 411001 | 3201001 | 8838800 | 8838802 | 8847138 |
|  | 8847765 | 8847809 | 8847867 |  |  |  |

These codes were defined based on the Various Information of Medical Fee operated by the Ministry of Health, Labour and Welfare (https://shinryohoshu.mhlw.go.jp/).

MRCNS, methicillin-resistant coagulase-negative staphylococci.
