# Supplementary material for: Influence of pharmacists and infection control teams or antimicrobial stewardship teams on the safety and efficacy of vancomycin: A Japanese administrative claims database study
Source: PLoS One. 2022 Sep 9;17(9):e0274324. doi: 10.1371/journal.pone.0274324 (PMC9462795; doi:10.1371/journal.pone.0274324)
Supplement: Attachment — Submitted filename: Response to Reviewers_revise.docx [file pone.0274324.s004.docx]

July 15, 2022

Emily Chenette

Editor-in-Chief

*PLoS ONE*

Thank you very much for reviewing our manuscript (PONE-D-22-09316) and providing us with the feedback and comments from the reviewers. We have received a number of comments from the three reviewers regarding patient background, various definitions, methods, and consistency between the title and the manuscript. We have reanalyzed and revised the text extensively following these comments. The changes in the revised manuscript have been shown in red font. We would appreciate your reviewing the manuscript again. We hope you find our revised manuscript suitable for publication in *PLoS ONE*.

**Response to Reviewer #1**

Thank you very much for taking the time to review our paper. We appreciate your helpful suggestions on many issues, including adjustments to the patient background, various definitions and biases, and the organization of the manuscript. In response to your comments, we have revised the title and content of the paper substantially. Our corrections and comments are as follows, and are indicated in red font in the revised manuscript. We would appreciate if you could confirm them.

Major comments:

***Comment 1***

*The retrospective study investigated the implementation of TDM over time and the effect of TDM on AKI and mortality. However, there has been relative solid evidence that therapeutic drug monitoring of vancomycin is associated with decreased risk of acute kidney injury. The rationale to perform this study requires further clarification.*

*The Introduction section stressed that the impact of pharmacist and ICT/AST intervention on VCM’s prognosis and side effects hasn’t been clarified. However, the study didn’t clarify this issue either, as I didn’t find that the study considered the intervention of pharmacists and infectious disease-associated teams adequately (only table 1 and post-hoc analysis on 30-day mortality mentioned intervention of pharmacists and infectious diseases-associated teams).*

**Response 1:**

As the reviewer pointed out, we have added the issues with the previous reports and further clarified the objectives of our study. In addition, we have revised the manuscript to focus on the influence on AKI and 30-day mortality.

**Change**: Introduction (Pages 5-6)

Please refer to the Introduction section of the revised manuscript.

***Comment 2***

*Line 102-105: The rationale of substitution TDM with treatment and management fee is not justified adequately, which may be vulnerable from misclassification bias. I was wondering could you provide more details on this issue, including how pharmacist and infectious disease – associated team intervention was identified.*

**Response 2:**

The treatment and management fee for a specific drug is a medical fee that can be calculated when TDM is performed. The method of replacing TDM with this fee was cited from several reports ^1-3)^. However, as the reviewer pointed out, there is a possibility of misclassification bias by substitution. Therefore, a description of misclassification bias has been added to the limitation.

Ref ^1)^: Imai S. et al. Association of the ward pharmacy service with active implementation of therapeutic drug monitoring for vancomycin and teicoplanin-an epidemiological surveillance study using Japanese large health insurance claims database. J Pharm Health Care Sci. 2020;6: 18.

Ref ^2)^: Saki Ito. et al. A study of trends and factors associated with therapeutic drug monitoring (TDM) implementation for arbekacin treatment using a large Japanese medical claims database. J Infect Chemother. 2022;28: 1266-1272.

Ref ^3)^: Takayuki M. et al. Implementation status of voriconazole therapeutic drug monitoring: analysis of data from a large Japanese health insurance claims database. [Japanese] TDMkenkyu. 2021;38: 39-48.

**Appendix**: Discussion (Page 19, Line 293)

There is a possibility of misclassification bias due to the substitution of reimbursement for TDM and selection bias due to the exclusion of patients receiving drugs that require TDM other than VCM.

***Comment 3***

*Figure 1: Please check the accuracy of number in Fig 1 again, especially in the first 2 steps.*

**Response 3:**

As you indicated, there was an error in the number of people excluded for the first two steps in Figure 1, so we have corrected it. Additionally, we have reviewed the paper again to see if there were any other mistakes. There were no other mistakes. We will be careful not to make such mistakes in the future.

We also received a comment from reviewer #2 for the exclusion criteria. As a result, we removed N18X from the definition of patients who were AKI prior to the index date, and re-extracted the population. Therefore, there have been other changes in Figure 1 as well.

**Change**: Fig 1

Please refer to Fig 1.

***Comment 4***

*The language of this manuscript requires major improvement, especially in Abstract section.*

**Response 4:**

Thank you very much for your comments. We have reviewed the content of all the submitted files, including the manuscript, and have again availed the English proofreading service.

Minor comments:

*Abstract:*

***Comment 1***

*The Introduction was overlong, and I don’t think that the contents of this study took account of interventions of pharmacists and infectious disease-associated teams adequately.*

**Response 1:**

Thank you very much for your comments. As the reviewer pointed out, we have revised the introduction to match the content of the text. Also, we have made the description brief.

**Before**: Introduction (Page 3, Line 23)

Nephrotoxicity is more likely to occur at high trough levels of vancomycin (VCM); therefore, therapeutic drug monitoring (TDM) is recommended. Infection control and appropriate use of antimicrobial agents have been promoted in hospitals by pharmacists and infectious disease-associated teams. This study aimed to evaluate the impact of TDM on the side effects and prognosis of VCM treatment using information from a claims database while considering the interventions of pharmacists and infectious disease-associated teams.

**Change**: Abstract introduction (Page 3, Line 27)

Methicillin-resistant Staphylococcus aureus (MRSA) has a high mortality and requires effective treatment with anti-MRSA agents such as vancomycin (VCM). Management of the efficacy and safety of VCM has been implemented with the assignment of pharmacists in hospital wards and the establishment of teams related to infectious diseases. However, there are no reports evaluating the association between these factors and the efficacy and safety of VCM in large populations.

***Comment 2***

*Methods: “The Japanese administrative claims database was used to evaluate the incidence of acute kidney injury (AKI) and 30-day mortality after VCM administration from 2010 to 2019 based on TDM implementation” I was wondering why the trend of acute kidney injury incidence and 30-day mortality from 2010 to 2019 was set as the primary analysis, which didn’t match to the title of this manuscript.*

**Response 2:**

We have revised the content, including the methods to focus on the risk factors for AKI and 30-day mortality and be better aligned with the study objectives. Also, the title has been changed to match the manuscript.

**Before**: Method (Page 3, Line 30)

The Japanese administrative claims database was used to evaluate the incidence of acute kidney injury (AKI) and 30-day mortality after VCM administration from 2010 to 2019 based on TDM implementation, which was assessed by calculating the treatment and management fee for specific drugs. The clinical variables that affected 30-day mortality were derived using logistic regression.

**Change**: Method (Page 3, Line 33)

This study used the Japanese administrative claims database accumulated from 2010 to 2019. The population was divided into two groups, therapeutic drug monitoring (TDM) group and non-TDM group, and adjusted by propensity score matching. We performed multivariate logistic regression analysis to determine the influence of pharmacists and infection control teams or antimicrobial stewardship teams on acute kidney injury (AKI) and 30-day mortality.

**Before**: Title (Page 1, Line 3)

Effect of therapeutic drug monitoring on the incidence of acute kidney injury and 30-day mortality after vancomycin administration: a Japanese administrative claims database study

**Change**: Title (Page 1, Line 3)

Influence of pharmacists and infection control teams or antimicrobial stewardship teams on the safety and efficacy of vancomycin: a Japanese administrative claims database study

***Comment 3***

*Results: More details (number, rate, ratio) of quantitative analysis should be added, rather than narrative description alone.*

**Response 3:**

Thank you very much for your comments. As the reviewer pointed out, we have added ORs and 95% CIs.

**Before**: Result (Page 3, Line 35)

The total number of patients was 70 768 (TDM group, n = 53 354; non-TDM group, n = 17 414). The incidence of AKI decreased over time in the TDM group. The 30-day mortality was lower in the TDM group than in the non-TDM group throughout the 10-year period. In addition, the study showed that large number of hospital beds, TDM implementation, and interventions by pharmacists and infectious disease-associated teams decreased 30-day mortality.

**Change**: Result (Page 3, Line 39)

The total number of patients was 73 478 (TDM group, n=55 269; non-TDM group, n=18 209). After propensity score matching, 18 196 patients were matched in each group. Multivariate logistic regression analysis showed that pharmacological management for each patient contributed to the reduction of AKI (odds ratio [OR]: 0.812, 95% confidence interval [CI]: 0.723‒0.912) and 30-day mortality (OR: 0.538, 95% CI: 0.503‒0.575). However, the establishment of infectious disease associated team in facilities and the assignment of pharmacists in the hospital wards had no effect on AKI and 30-day mortality. In addition, TDM did not affect the reduction in AKI (OR: 1.061, 95% CI: 0.948‒1.187), but reduced 30-day mortality (OR: 0.873, 95% CI: 0.821‒0.929).

*Introduction:*

***Comment 4***

*Line 84-86: I get it that the study at first investigated the implementation of vancomycin TDM over time, and then evaluated the effect of TDM on mortality and AKI. However, the consideration of the interventions of pharmacists and infectious disease-associated teams was inadequate. Could you please rephrase this part?*

**Response 4:**

According to the reviewers' comments, we have revised the text to focus on AKI and 30-day mortality, and have revised the description of the objectives to be more appropriate for the content.

**Before**: Introduction (Page 7, Line 84)

We evaluated the impact of TDM on the side effects and prognosis related to VCM treatment using a claims database, while considering the interventions of pharmacists and infectious disease-associated teams.

**Change**: Introduction (Page 6, Line 83)

In this study, we examined the factors affecting the efficacy and safety of VCM, while considering the interventions of pharmacists, ICTs and ASTs.

*Methods:*

***Comment 5***

*Line 152-157: The primary analysis didn’t match with the objective/title of this study. I was wondering why the linear regression was performed on the trend of AKI incidence and 30-day mortality in both TDM and non-TDM group.*

*Rather, the multivariable logistic regression was used as post-hoc analysis. I am not sure whether the authors adjust confounders properly.*

*I suggest to add the post-hoc multivariable analysis on AKI.*

**Response 5:**

We have revised the content to focus on AKI and 30-day mortality. As the reviewer pointed out, we performed propensity score matching on variables related to TDM implementation and adjusted for patient background. We then performed a multivariate analysis for AKI and 30-day mortality.

**Before**: Method - Statistical analysis (Page 10, Line 149)

The patient characteristics were analyzed descriptively, and the rate of TDM implementation was evaluated using the Cochran‒Amitage trend test. Descriptive data were expressed as median (interquartile range), numbers, and percentages.

High-dose VCM is a risk factor for nephrotoxicity [21]; therefore, the dose was set as a process measure, and based on previous reports, linear regression was performed on the median to evaluate the trend over time [6]. Similarly, we set the incidence of AKI and 30-day mortality as outcome measures and performed linear regression. In addition, the chi-square test was performed to compare the incidence of outcomes between the TDM and non-TDM groups. In the post-hoc analysis, multivariate logistic regression analysis was performed for 30-day mortality using sex, age, number of beds, treatment category, CCI, initial daily dose/weight, various reimbursements, and site of infection as independent variables after applying the chi-square test or Mann‒Whitney U test, as appropriate.

All statistical analyses were performed using the Stata software, version 17.0 (Stata Corp., College Station, TX); the significance level was set at p < 0.05.

**Change**: Method - Statistical analysis (Page 10, Line 145)

Propensity scores were calculated by logistic regression analysis and 1:1 matching was performed with a caliper distance of 0.2. In this study, propensity score matching was performed using the following variables as factors related to the implementation of TDM; number of beds, CCI, infectious disease-associated teams fee, inpatient pharmaceutical service premium, respiratory infection, bacteremia/sepsis, urinary tract infections, intra-abdominal infections, skin and soft tissue infections, bone and joint infections, central nervous system infections, febrile neutropenia, infective endocarditis, and MRCNS infection. The variable balance between the two groups after propensity score matching was evaluated by standardized mean difference (SMD). A SMD < 0.10 suggests an appropriate variable balance. In addition, univariate and multivariate logistic regression analyses were performed with each as the objective variable in order to clarify the risk factors for AKI and 30-day mortality.

Statistical analysis was performed using Stata software, version 17.0 (Stata Corp., College Station, TX) and EZR (Saitama Medical Center, Jichi Medical University, Saitama, Japan) [17], with a significance level set at p < 0.05.

*Results:*

***Comment 6***

*Table 1: I was wondering could you please add the baseline characteristics of patients in the TDM group and non-TDM group, which may help interpret the results.*

**Response 6:**

Thank you very much for your comments. As the reviewer pointed out, after propensity score matching was performed, we have indicated the baseline characteristics of each group in Table 1.

**Change**: Table 1 (Page 12, Line 183)

Please refer to Table 1.

***Comment 7***

*Line 207: I didn’t get it why the initial VCM dose was analyzed, as initial VCM dose may be less relevant to TDM implementation. The change and maintain dose following TDM may be more clinically relevant.*

**Response 7:**

After receiving the comments of the three reviewers, we reviewed the structure of the text and our analysis. As a result, we have refrained from mentioning this point.

**Before**: Result - TDM implementation rate and VCM dose (Page 16, Line 207)

Table 2. The change in trend of the initial VCM dose with TDM from 2010 to 2019

**Change**: This has been deleted form the revised manuscript.

***Comment 8***

*Line 238: “increased” should be removed from the table title.*

**Response 8:**

Thank you for pointing this out. As the reviewer pointed out, we have removed the word "increased".

**Before**: Result - Identifying factors affecting 30-day mortality (Page 18, Line 238)

Table 3. Clinical variables associated with increased 30-day mortality

**Change**: Result - Identifying factors affecting 30-day mortality (Page 16, Line 232)

Table 3. Clinical variables associated with 30-day mortality in the adjusted population

***Comment 9***

*The results section should be re-organized and make it more logically.*

**Response 9:**

After receiving comments from the three reviewers, we have extensively reanalyzed and revised the the result section.

**Change**: Result

Please refer to Result.

*Discussion:*

***Comment 10***

*Line 261-262: the unit should be μg/mL*

**Response 10:**

As with Response 7, we have avoided mentioning this point.

**Before**: Discussion (Page 24, Line 261)

Increased dosing has led to the adjustment of trough values to 10‒15 µg [24] or 15‒20 µg [25].

**Change**: This has been deleted form the revised manuscript.

***Comment 11***

*Line 284-285: I am not sure whether the study assessed “TDM and interventions by pharmacists and infectious disease-associated teams” as a whole.*

**Response 11:**

After the reviewer's comments, we have revised the text to focus on AKI and 30-day mortality. As a result, after adjusting for patient background based on the TDM implementation, we analyzed the influence of the intervention by the pharmacist and the infectious disease-related team. We would appreciate your review again.

***Comment 12***

*Did the study adjust for measured confounders properly? Also, pleased provide examples of important unmeasured confounders*

*Please add potential selection bias (Patients who received drugs requiring TDM other than VCM within 7 days of the index date were excluded)*

*I am not sure whether the method of using reimbursement to identify TDM-related intervention would be vulnerable from misclassification bias.*

*One of the strengths of claim data may be long follow-up, which allow us to assess TDM on 3-day mortality.*

**Response 12:**

As the reviewer pointed out, we performed propensity score matching and adjusted for patient background. We have also added severity of infection and history of surgery to the limitation section as examples of important unmeasured confounders. In addition, the possibility of selection bias has been described in the limitations.

**Appendix**: (Page 19, Line 293)

There is a possibility of misclassification bias due to the substitution of reimbursement for TDM and selection bias due to the exclusion of patients receiving drugs that require TDM other than VCM. These definitions may underestimate TDM implementation.

**Appendix**: (Page 20, Line 299)

Finally, there were unmeasured confounders including severity of infection and surgical history.

**Response to Reviewer #2**

Thank you very much for taking the time to review this paper. We appreciate your helpful suggestions on adjusting for patient background, defining AKI, and describing limitations. In response to many of the comments from you and other reviewers, we have made significant revisions to the content. Our corrections and comments are as follows, and are indicated in red font in the revised manuscript. We would appreciate it if you could confirm them.

Major comments:

***Comment 1***

*Are the diagnosis names of AKI (e.g., ICD-10 codes) validated? Do these AKI diagnostic names reflect true AKI? The percentage of AKI in this study is clearly lower than in previous reports.*

**Response 1:**

In previous database reports, the ICD-10 code N17X has been used as the definition of AKI ^1,2)^. However, as the reviewer pointed out, AKI incidence was lower in this report than in the previous reports. Therefore, we have cited a report on the validation of the sensitivity of defining AKI using ICD-10 ^3)^ and discussed the possibility of underestimating AKI.

Ref ^1)^: Ikuta K. et al. Association of proton pump inhibitors and concomitant drugs with risk of acute kidney injury: a nested case-control study. BMJ Open. 2021;11 :e041543.

Ref ^2)^: Mohamed TH. et al. Nephrotoxic medications and associated acute kidney injury in hospitalized neonates. J Nephrol. 2022. Epub ahead of print.

Ref ^3)^: Hwang J. et al. Validity of the International Classification of Diseases, Tenth Revision code for acute kidney injury in elderly patients at presentation to the emergency department and at hospital admission. BMJ Open. 2012;2: e001821.

**Appendix**: Method - Definitions and clinical variables (Page 9, Line 134)

A previous study in elderly subjects had shown that ICD-10 code N17X for AKI had moderate sensitivity [16].

**Appendix**: Discussion (Page 20, Line 296)

Similarly, AKI was substituted for ICD-10 code and Japanese disease code. The incidence of AKI may also have been underestimated. In addition, the timeline of AKI diagnosis and VCM administration may have been reversed.

***Comment 2***

*How did the authors handle suspected diagnosis? Clinicians often make a suspected diagnosis to levy medical fees for performing blood tests and prescriptions in Japan.*

**Response 2:**

Thank you very much for your comments. AKI was determined in patients with a confirmed diagnosis. Therefore, patients with only a suspected diagnosis were not extracted. We have added this point in the text.

**Appendix**: Methos - Definitions and clinical variables (Page 9, Line 138)

The data of only the patients with a confirmed diagnosis of AKI was extracted, and patients with only a suspected diagnosis were not included.

***Comment 3***

*Why did the authors exclude CKD patients (N18X)?*

**Response 3:**

Initially, we thought it would be difficult to evaluate AKI in patients who already have renal impairment, as in CKD. However, even patients with CKD have variable renal function and may develop acute kidney injury. Therefore, we removed CKD from the exclusion criteria and performed the analysis again.

**Before**: Method – Study population (Page 8, Line 114)

For exclusion, nephrotoxicity was defined based on the International Classification of Diseases 10th version (ICD-10) codes (N17X: acute renal failure, N18X: chronic kidney disease) and five Japanese disease codes (5839017: kidney injury, 8835584: reduced renal function, 8840719/9952036: drug-induced kidney injury, 8849597: acute kidney injury [AKI]).

**Change**: Method – Study population (Page 9, Line 131)

AKI was defined based on the International Classification of Diseases 10th version (ICD-10) codes (N17X: acute renal failure) and five Japanese disease codes (5839017: kidney injury, 8835584: reduced renal function, 8840719/9952036: drug-induced kidney injury, 8849597: acute kidney injury [AKI]).

***Comment 4***

*Usually, there is a difference in patient background between patients with TDM and those without. The authors should confirm this difference and then evaluate outcomes after adjusting for patient background (e.g., propensity score matching).*

**Response 4:**

As the reviewer pointed out, we performed propensity score matching on variables related to TDM implementation and adjusted for patient background.

**Before:** Method – Statistical analysis (Page 10, Line 149)

The patient characteristics were analyzed descriptively, and the rate of TDM implementation was evaluated using the Cochran‒Amitage trend test. Descriptive data were expressed as median (interquartile range), numbers, and percentages.

High-dose VCM is a risk factor for nephrotoxicity [21]; therefore, the dose was set as a process measure, and based on previous reports, linear regression was performed on the median to evaluate the trend over time [6]. Similarly, we set the incidence of AKI and 30-day mortality as outcome measures and performed linear regression. In addition, the chi-square test was performed to compare the incidence of outcomes between the TDM and non-TDM groups. In the post-hoc analysis, multivariate logistic regression analysis was performed for 30-day mortality using sex, age, number of beds, treatment category, CCI, initial daily dose/weight, various reimbursements, and site of infection as independent variables after applying the chi-square test or Mann‒Whitney U test, as appropriate.

**Change**: Method – Statistical analysis (Page 10, Line 145)

Propensity scores were calculated by logistic regression analysis and 1:1 matching was performed with a caliper distance of 0.2. In this study, propensity score matching was performed using the following variables as factors related to the implementation of TDM; number of beds, CCI, infectious disease-associated teams fee, inpatient pharmaceutical service premium, respiratory infection, bacteremia/sepsis, urinary tract infections, intra-abdominal infections, skin and soft tissue infections, bone and joint infections, central nervous system infections, febrile neutropenia, infective endocarditis, and MRCNS infection. The variable balance between the two groups after propensity score matching was evaluated by standardized mean difference (SMD). A SMD < 0.10 suggests an appropriate variable balance. In addition, univariate and multivariate logistic regression analyses were performed with each as the objective variable in order to clarify the risk factors for AKI and 30-day mortality.

Minor comments:

***Comment 1***

*Figure 1 has two calculation errors*

**Response 1:**

As you indicated, there was an error in the number of people excluded for the first two steps in Figure 1, so we have entered the correct values. We have further reviewed the paper repeatedly for any other mistakes, and confirmed that there were no other mistakes. We will be careful not to make such mistakes in the future. Also, following your major comment 3, we have removed N18X from the definition of patients who were diagnosed with AKI prior to the index date, and re-extracted the population. Therefore, there are other changes in Figure 1 besides the first two steps.

**Change**: Fig. 1

Please refer to Fig 1.

***Comment 2***

*The limitation is inadequate. For example, a discussion of sampling bias resulting from the employing DPC data is needed. The authors are only evaluating prescriptions, not the actual dosing situation.*

**Response 2:**

In this study, there is a sampling bias that does not include all patients in Japan due to the use of DPC data. In addition, the data obtained is prescription information and may not reflect actual administration. These two points have been added to the limitation section.

**Before**: Discussion (Page 26, Line 298)

The first is exclusive to database studies — in some cases, data may be missing or the data entry format may not be standardized across facilities. Therefore, the incidence of AKI and dose may not be accurate.

**Change**: Discussion (Page 19, Line 287)

The first is the items in the database study. In some cases, data may be missing or the data entry format may not be consistent across facilities. Also, as we are unable to identify post-discharge outcomes, there is a possibility of underestimating 30-day mortality. In addition, this population may not be representative due to sampling bias because it does not cover all patients in Japan. The data obtained is prescription information and may not reflect actual dosing.

***Comment 3***

*The authors should recognize the difference between rate, proportion, and ratio and use them appropriately.*

**Response 3:**

We have reviewed the content of your comment and corrected the inappropriate use of "rate" to "proportion". The same has been confirmed by English proofreading.

**Before**: Discussion (Page 26, Line 296)

Bacteremia/sepsis is a serious infection with a mortality rate of over 25‒30% [30] and may be a factor associated with increased 30-day mortality rates.

**Change**: Discussion (Page 18, Line 271)

Bacteremia/sepsis is a serious infection with a mortality proportion of over 25‒30% [25] and may be a factor associated with increased 30-day mortality.

**Response to Reviewer #3**

We appreciate your helpful comments on the definition of AKI and limitations in this database. After receiving lots of comments from you and other reviewers, we have redone the analysis from patient extraction and revised the content significantly. Our corrections and comments are as follows, and shown in red font. We would appreciate it if you could confirm them.

Major comments:

***Comment 1***

*Authors defined for AKI as primally endpoint such as “AKI was diagnosed based on the ICD-144 10 code (N17X) and five Japanese disease codes (5839017: kidney injury, 8835584:145 reduced renal function, 8840719/ 9952036: drug-induced kidney injury, 8849597: AKI).”*

*Definition for AKI using N17X is reported for inadequate by the results of validation analysis in renal transplantation patients 1, 2) In addition, Japanese disease codes for authors definition may not be adequate to detect AKI.*

*Ref 1) JSPE committee, reports available from “https://www.jspe.jp/committee/pdf/*

*validationtrr120180523.pdf” (see p.27, line 16)*

*Ref 2) Amber O. Molnar etal. Validation of administrative database codes for acute kidney injury in kidney transplant recipients. Can J Kidney Health Dis. 2016; 3: 18.*

**Response 1:**

In previous database reports, the ICD-10 code N17X has been used as the definition of AKI ^1,2)^. However, as the reviewer pointed out, the ICD-10 code for AKI has been reported to be insufficiently sensitive in renal transplant patients. On the other hand, there is a report that validated the sensitivity using N17X in an elderly population that is more similar to the patient population of our study ^3)^. Therefore, we have cited this article and added to the methods and limitations of our study. We have also added five disease codes because we thought they might be necessary for drug-induced kidney injury in VCM. However, since AKI may be underestimated even after using this definition, we have added a further limitation.

Ref 1): Ikuta K. et al. Association of proton pump inhibitors and concomitant drugs with risk of acute kidney injury: a nested case-control study. BMJ Open. 2021;11 :e041543.

Ref 2): Mohamed TH. et al. Nephrotoxic medications and associated acute kidney injury in hospitalized neonates. J Nephrol. 2022. Epub ahead of print.

Ref 3): Hwang J. et al. Validity of the International Classification of Diseases, Tenth Revision code for acute kidney injury in elderly patients at presentation to the emergency department and at hospital admission. BMJ Open. 2012;2: e001821.

**Appendix**: Method - Definitions and clinical variables (Page 9, Line 134)

A previous study in elderly subjects had shown that ICD-10 code N17X for AKI had moderate sensitivity [16].

**Appendix**: Discussion (Page 20, Line 296)

Similarly, AKI was substituted for ICD-10 code and Japanese disease code. The incidence of AKI may also have been underestimated.

***Comment 2***

*Detailed date for AKI is able to define by using DPC data? Is the time axis reversed between AKI diagnosis and VCM administration?*

**Response 2:**

Thank you very much for your comments. As the reviewer pointed out, we cannot rule out the possibility that the time axis is reversed. Therefore, we have handled this issue as much as possible by excluding patients with AKI in the month prior to the index date. This point has been added to the limitation.

**Appendix**: Discussion (Page 20, Line 297)

In addition, the timeline of AKI diagnosis and VCM administration may have been reversed.

***Comment 3***

*How to analyze “30-day mortality after VCM administration.”? Authors can assess death after the discharge? Administrative data from Medical Data Vision can provide DPC (in-hospital) and the after discharge?*

**Response 3:**

Thank you very much for your comments. As the reviewer pointed out, we cannot track outcomes after discharge. We have added this point to the limitation.

**Appendix**: Discussion (Page 19, Line 289)

Also, as we are unable to identify post-discharge outcomes, there is a possibility of underestimating 30-day mortality.

Minor comments:

***Comment 1***

*Authors calculated “rate of TDM implementation” Is it correct? Is it proportion? (line 149)*

**Response 1:**

Thank you very much for your comments. As the reviewer pointed out, "proportion" was appropriate. However, we have removed this part of the text in the revision.

**Before**: Method – Statistical analysis (Page 10, Line 149)

The patient characteristics were analyzed descriptively, and the rate of TDM implementation was evaluated using the Cochran‒Amitage trend test. Descriptive data were expressed as median (interquartile range), numbers, and percentages.

**Change**: This has been deleted form the revised manuscript.
